# Incidence and Comparative Risk of Hematological Adverse Events Across Different Immune Checkpoint Inhibitors: A Network Meta-Analysis of Randomized Controlled Trials

**DOI:** 10.7759/cureus.105307

**Published:** 2026-03-16

**Authors:** Jaahnavi Vajje, Nafisa Reyaz, Iman Gillani, Muhammad Muneeb Ullah, Sandipkumar S Chaudhari, Sidra Jabeen, Calvin R Wei, Adil Amin

**Affiliations:** 1 Internal Medicine, Dr.Pinnamaneni Siddhartha Institute of Medical Sciences and Research Foundation, Vijayawada, IND; 2 Medicine, Jawaharlal Nehru Medical College and Hospital, Aligarh, IND; 3 Medical Education, Sheikh Khalifa Bin Zayed Al-Nahyan University, Lahore, PAK; 4 Medicine and Surgery, Rashid Latif Medical College, Lahore, PAK; 5 Cardiothoracic Surgery, University of Alabama at Birmingham, Birmingham, USA; 6 Family Medicine, University of North Dakota School of Medicine and Health Sciences, Fargo, USA; 7 Internal Medicine, Sancti Spiritus, CUB; 8 Research and Development, Shing Huei Group, Taipei, TWN; 9 Cardiology, Pakistan Navy Ship Shifa, Karachi, PAK

**Keywords:** cancer immunotherapy, drug safety, hematological adverse events, immune checkpoint inhibitors, network meta-analysis

## Abstract

Cancer immunotherapy with immune checkpoint inhibitors (ICIs) has transformed oncologic treatment, yet hematological adverse events (HAEs) remain incompletely characterized across different agents. We conducted a systematic review and network meta-analysis to compare the incidence and risk of hematological toxicities (all grades) among various ICIs. A comprehensive literature search was performed across PubMed, EMBASE, Cochrane Central Register of Controlled Trials, and Web of Science through January 2026. Phase II and III randomized controlled trials (RCTs) evaluating ICIs and reporting HAEs were included. Frequentist network meta-analysis was performed to estimate odds ratios and rankings using surface under the cumulative ranking curve values for anemia, neutropenia, and thrombocytopenia. Thirty-seven RCTs encompassing diverse malignancies were included in the network meta-analysis. For anemia, ipilimumab demonstrated the most favorable safety profile, followed by toripalimab and pembrolizumab, while serplulimab and nivolumab ranked lowest. Regarding neutropenia, ipilimumab again showed the best safety ranking, followed by avelumab and pembrolizumab, whereas toripalimab and serplulimab exhibited less favorable profiles. For thrombocytopenia, ipilimumab ranked highest, followed by sintilimab and nivolumab, while toripalimab showed the least favorable ranking. No significant global inconsistency was detected across networks. Publication bias was identified for neutropenia but not for anemia or thrombocytopenia. This network meta-analysis reveals substantial heterogeneity in hematological safety profiles across ICIs, with ipilimumab consistently demonstrating favorable rankings across all HAEs. These findings suggest potential differences in hematologic safety profiles across treatments. However, given the star-shaped network structure, reliance on indirect comparisons, and observed heterogeneity, the results should be interpreted cautiously. While not definitive, the analysis may still offer preliminary insights to inform treatment selection, particularly for patients with baseline hematological vulnerabilities or those receiving concurrent myelosuppressive therapies, and may help guide the development of risk-stratified monitoring strategies in clinical practice.

## Introduction and background

Cancer continues to be one of the foremost causes of death globally, with approximately 20 million new diagnoses and 9.7 million cancer-related deaths reported in 2022 [1]. In recent years, immune checkpoint inhibitors (ICIs) have transformed the landscape of oncology by delivering substantial survival improvements across a wide range of tumor types [2]. These therapies, most notably inhibitors targeting programmed death-1 (PD-1), programmed death-ligand 1 (PD-L1), and cytotoxic T-lymphocyte-associated protein 4 (CTLA-4), enhance antitumor immunity by disrupting inhibitory immune signaling pathways [3]. Nevertheless, the immune activation induced by these agents is accompanied by a spectrum of immune-related adverse events (irAEs) that may involve virtually any organ system [4].

Among these toxicities, hematological adverse events (HAEs) are relatively underrecognized but carry important clinical implications, particularly with respect to treatment tolerability and continuity [5]. Reported manifestations include common cytopenias such as anemia, thrombocytopenia, and neutropenia, as well as rarer but potentially life-threatening conditions, including immune-mediated thrombocytopenic purpura, autoimmune hemolytic anemia, and aplastic anemia [6-7]. The incidence of hematological toxicities associated with ICIs varies widely across studies, with reported rates ranging from approximately 1% to 15%, influenced by the specific agent used and the underlying malignancy [8]. Although many cases are manageable, severe hematological complications may necessitate treatment delays, dose adjustments, or permanent discontinuation, thereby potentially diminishing therapeutic benefit [9].

HAEs associated with ICIs were evaluated as all-grade events in the current study to capture the full spectrum of toxicity across treatments. Although many HAEs may be mild, they can still carry clinically relevant consequences, including the need for blood transfusions, increased susceptibility to infections, treatment delays or discontinuation, and additional monitoring requirements [7]. Understanding the comparative risk of these events across agents is therefore important for informing treatment selection and optimizing patient management in clinical practice.

Despite the growing number of ICIs approved for clinical use, comparative data assessing their relative hematological safety remain limited [10]. Existing evidence largely originates from single-arm studies or trials comparing ICIs with conventional chemotherapy, offering little direct guidance for clinicians when choosing among different checkpoint inhibitors [11]. This lack of comparative safety data is increasingly concerning as ICIs are incorporated into combination strategies and deployed earlier in the course of disease across diverse cancer populations [12].

Network meta-analysis (NMA) provides a robust analytical framework to overcome this challenge by facilitating indirect comparisons among interventions that have not been directly evaluated against one another in randomized controlled trials (RCTs) [13]. By integrating all available evidence within a unified statistical model, NMA allows for the estimation of relative risks and the ranking of treatments based on specific outcomes [14]. This methodology has been widely adopted in multiple therapeutic fields to support evidence-based clinical decision-making [15].

In light of the limited head-to-head evidence and the clinical relevance of hematological toxicity during ICI therapy, we performed a systematic review and NMA to evaluate and compare the incidence and relative risk of HAEs across different ICIs. The findings of this study are intended to provide clinicians and patients with high-quality comparative safety evidence to support informed treatment selection and balanced risk-benefit discussions in contemporary oncology practice.

## Review

Methodology

Literature Search

A systematic and exhaustive literature search was undertaken in compliance with the Preferred Reporting Items for Systematic Reviews and Meta-Analyses extension for Network Meta-Analysis (PRISMA-NMA) guidelines [16]. Four electronic bibliographic databases - PubMed/MEDLINE, EMBASE, the Cochrane Central Register of Controlled Trials (CENTRAL), and the Web of Science Core Collection - were comprehensively searched. All relevant records published from database inception up to 31/01/2026 were considered eligible. The protocol was registered in PROSPERO (CRD43024648217).

Search Strategy

A predefined and structured search strategy was developed using a combination of controlled vocabulary terms (e.g., Medical Subject Headings) and text-based keywords. Search terms were organized around three core concepts: (1) ICI therapies, including individual agents such as pembrolizumab, nivolumab, atezolizumab, durvalumab, avelumab, ipilimumab, cemiplimab, and dostarlimab, as well as broader descriptors including “PD-1 inhibition,” “PD-L1 blockade,” “CTLA-4 inhibition,” and “immune checkpoint therapy”; (2) hematological toxicities, incorporating terms such as “hematologic adverse events,” “cytopenia,” “anemia,” “neutropenia,” “thrombocytopenia,” “leukopenia,” and “pancytopenia”; and (3) methodological filters designed to identify RCTs. Boolean operators (AND/OR) were applied to combine search terms appropriately. No language restrictions were imposed during the database searches. In addition, the reference lists of all included studies and relevant review articles were manually examined to identify further eligible trials not captured through electronic searching (detailed strategy in Appendix A).

Study Selection and Eligibility Criteria

All retrieved records were independently screened by two reviewers through a two-stage process involving title and abstract review, followed by a full-text assessment of potentially eligible articles. Any discrepancies were resolved through discussion and, when necessary, adjudication by a third senior reviewer. Studies were included if they met the following criteria: (1) prospective phase II or phase III RCTs enrolling adult patients with any type of malignancy; (2) evaluation of ICIs administered either as monotherapy or in combination with systemic anticancer treatments; (3) reporting extractable data on HAEs, specifically anemia, neutropenia, and/or thrombocytopenia; (4) publication as full-text articles in peer-reviewed journals; and (5) sufficient reporting of treatment arms and outcomes to allow incorporation into an NMA framework.

Studies were excluded for any of the following reasons: (1) non-randomized designs, including observational studies, case reports, case series, or single-arm trials; (2) phase I or dose-escalation studies; (3) duplicate reports or secondary analyses of previously published trials; (4) publications limited to conference abstracts or posters without a corresponding full-text article; (5) insufficient reporting of hematological toxicity data; or (6) trials comparing ICIs combined with identical background therapies in which the independent effect of the immunotherapy agent could not be clearly distinguished.

Data Extraction

A standardized data collection template was designed and pilot-tested prior to formal extraction. One reviewer extracted all relevant data, and a second reviewer independently cross-checked the extracted information to ensure accuracy and completeness. The following data were collected: (1) study-level characteristics, including first author, year of publication, trial phase, sample size, and duration of follow-up; (2) baseline patient characteristics such as median age, sex distribution, and cancer type; (3) treatment characteristics, including specific ICI agents and any concomitant therapies used in combination arms; (4) outcome data detailing the number of patients experiencing anemia, neutropenia, and thrombocytopenia; and (5) indicators related to methodological rigor and study quality. In studies with multiple intervention arms, only those arms relevant to the predefined network comparisons were included.

Quality Assessment and Risk of Bias

The methodological quality of all included RCTs was independently evaluated by two reviewers using the Cochrane Risk of Bias 2 (RoB 2) tool [17]. Five domains were assessed: bias arising from the randomization process, bias due to deviations from the intended interventions, bias related to missing outcome data, bias in outcome measurement, and bias in selective reporting of results. Each domain was categorized as low risk, some concerns, or high risk of bias based on established criteria. An overall risk of bias judgment was derived from the domain-level assessments. Potential publication bias was examined through visual inspection of comparison-adjusted funnel plots and, when ten or more studies were available for a given outcome, formally tested using Egger’s regression asymmetry test.

Outcomes

The primary endpoints of this NMA were the incidence and comparative risk of HAEs associated with different ICI regimens. Analyses were conducted for three prespecified hematological toxicities: anemia, neutropenia, and thrombocytopenia of all grades.

Data Analysis

NMA was performed using a frequentist framework with random-effects models to account for anticipated heterogeneity across studies. All statistical analyses were conducted using R software (version 4.3.0; R Foundation for Statistical Computing, Vienna, Austria) with the netmeta package. Risk ratios (RRs) with corresponding 95% confidence intervals (CIs) were calculated as the summary measure of treatment effect for each HAE outcome.

Statistical heterogeneity within each direct pairwise comparison was quantified using the I² statistic, which represents the percentage of variability attributable to between-study heterogeneity rather than sampling error. I² values of 25%, 50%, and 75% were interpreted as indicating low, moderate, and high heterogeneity, respectively. Between-study variance (τ²) was also estimated to quantify the extent of heterogeneity on the log odds ratio scale. The Q statistic and its associated p-value were calculated to test for the presence of statistically significant heterogeneity within each comparison.

The consistency assumption-that direct and indirect evidence are in agreement-was evaluated using a global approach. Global inconsistency across the entire network was assessed using the design-by-treatment interaction model, which tests whether treatment effects vary systematically across different study designs. The Q statistic for inconsistency was calculated along with degrees of freedom and corresponding p-values, with p < 0.05 indicating evidence of global inconsistency. Given the star-shaped configuration of the networks centered on control comparisons with limited or absent closed loops, local inconsistency assessment through node-splitting analysis was not feasible.

To facilitate clinical interpretation and inform treatment selection, all ICIs were ranked according to their safety profiles for each HAE using surface under the cumulative ranking curve (SUCRA) values. SUCRA provides a numerical summary ranging from 0% to 100%, with higher values indicating superior safety (lower risk of adverse events). A SUCRA value of 100% indicates certainty that a treatment is the safest option, while 0% indicates certainty that it is the least safe. SUCRA values were calculated for each treatment and outcome, and treatments were ranked in descending order of SUCRA percentages.

Results

**Figure 1:**
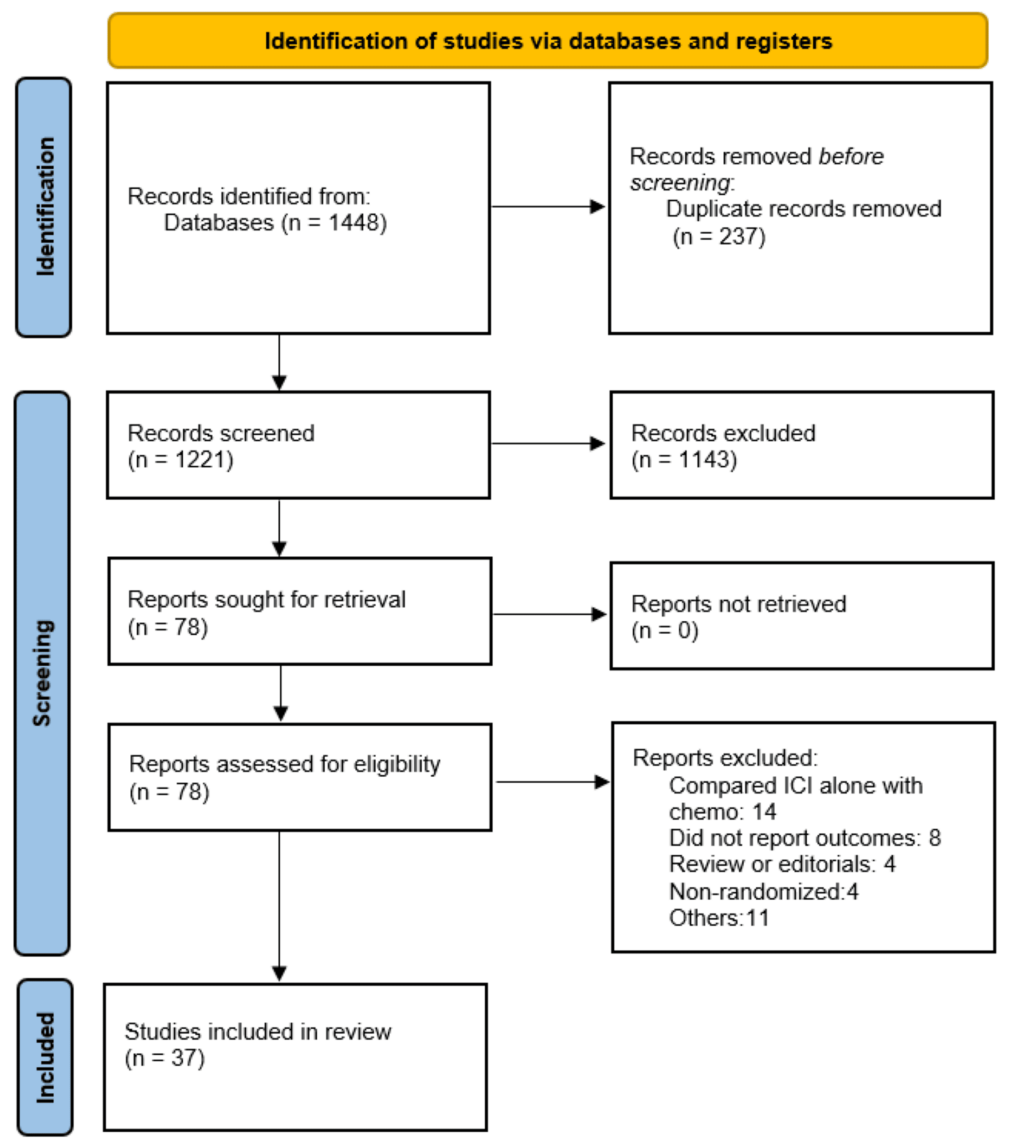
Study selection process

Using a comprehensive search strategy, a total of 1,448 records were initially identified. After removal of 237 duplicate citations, the remaining studies underwent title and abstract screening, resulting in 78 articles being retrieved for full-text evaluation. Following application of the predefined inclusion and exclusion criteria, 37 RCTs assessing nine distinct ICIs were ultimately included in the NMA. The study selection process is illustrated in the PRISMA flow diagram presented in Figure 1.

Characteristics of the Included Studies

**Figure 2:**
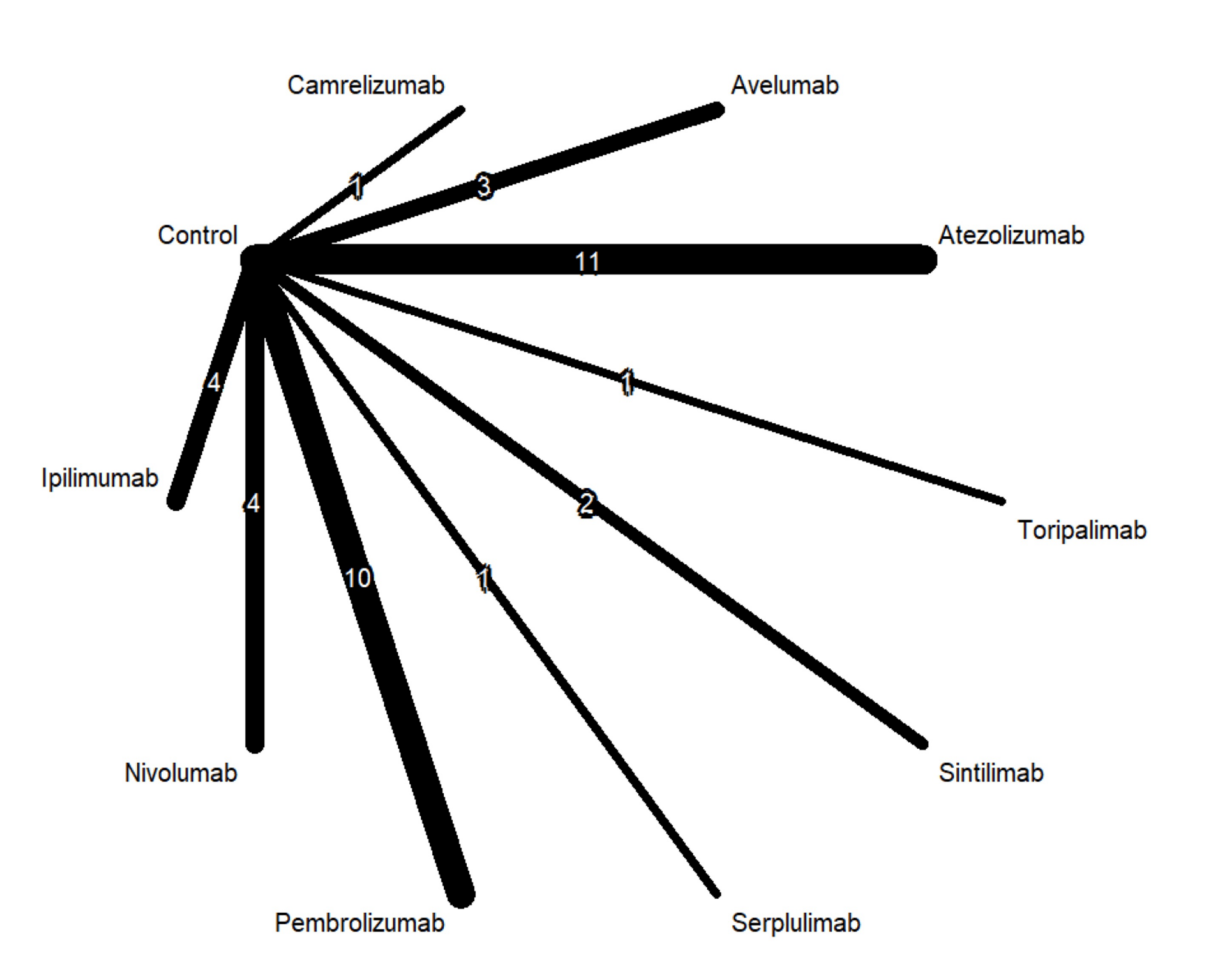
Network graph for anemia Image created by the authors with RStudio (Posit PBC, USA)

**Figure 3:**
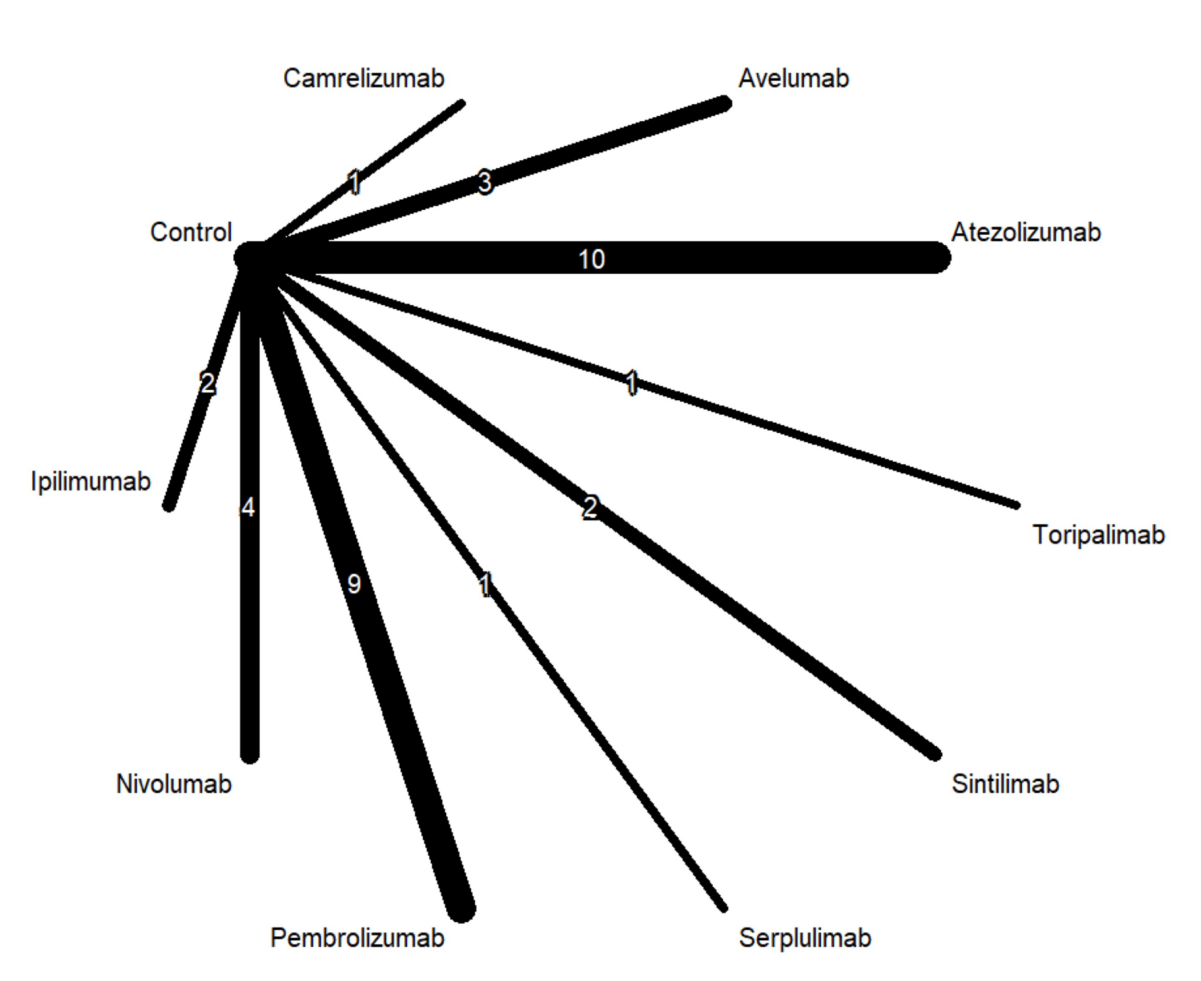
Network graph for neutropenia Image created by the authors with RStudio (Posit PBC, USA)

**Figure 4:**
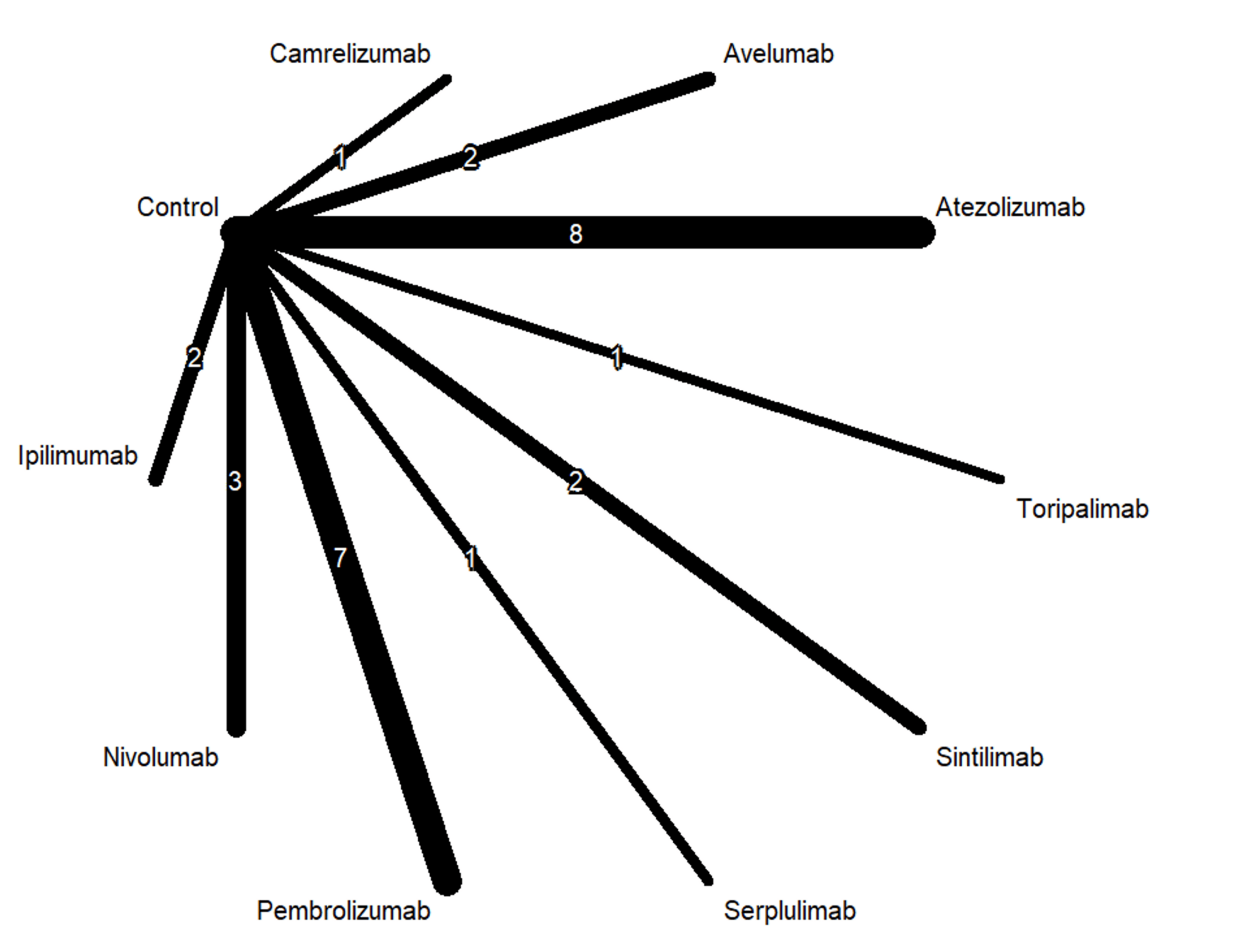
Network graph for thrombocytopenia Image created by the authors with RStudio (Posit PBC, USA)

Table 1 shows 37 RCTs published between 2010 and 2024. The types of cancer tested in these studies included esophageal cancer, gastric and biliary tract cancer, lung cancer, ovarian cancer, breast cancer, melanoma, head and neck and prostate cancer. The majority of the studies included ICI as the first-line treatment. Figures 2-4 show the network graphs illustrating comparisons for anemia, neutropenia, and thrombocytopenia, respectively. Table 3 presents the quality assessment of the included studies.

**Table 1:** Characteristics of the included studies

Study ID	Cancer	Study setting	Groups	Total
Cortes et al., 2020 [18]	Breast cancer	First-line	Pembrolizumab	562
			Control	281
Doki et al., 2022 [19]	Esophageal	First-line	Nivolumab	321
			Control	324
Galsky et al., 2020 [20]	Urothelial cancer	First-line	Atezolizumab	453
			Control	390
Gandhi et al., 2018 [21]	Lung cancer	First-line	Pembrolizumab	405
			Control	202
Govindan et al., 2017 [22]	Lung cancer	First-line or later	Ipilimumab	475
			Control	473
Gutzmer et al., 2020 [23]	Melanoma	First-line	Atezolizumab	230
			Control	281
Hodi et al., 2010 [24]	Melanoma	Second or later	Ipilimumab	380
			Control	132
Horn et al., 2018 [25]	Lung cancer	First-line	Atezolizumab	198
			Control	196
Janjigian et al., 2021 [26]	Gastric cancer	First-line	Nivolumab	782
			Control	767
Jotte et al., 2020 [27]	Lung cancer	First-line	Atezolizumab	334
			Control	334
Kang et al., 2022 [28]	Gastro	First-line	Nivolumab	362
			Control	362
Kelly et al., 2023 [29]	Biliary tract cancer	First-line	Pembrolizumab	529
			Control	534
Kwon et al., 2014 [30]	Prostate cancer	Second or later	Ipilimumab	393
			Control	396
Lee et al., 2021 [31]	Head and neck	First-line	Avelumab	348
			Control	344
Lu et al., 2022 [32]	Esophageal	First-line	Sintilimab	327
			Control	332
Luo et al., 2021 [33]	Esophageal	First-line	Camrelizumab	298
			Control	298
Mateos et al., 2019 [34]		Third or later	Pemb	122
	Myeloma		Control	123
Miles et al., 2021 [35]	Breast cancer	First-line	Atezolizumab	431
			Control	218
Mittendorf et al., 2020 [36]	Breast cancer	First-line	Atez	164
			Control	167
Moore et al., 2021 [37]	Ovarian cancer	Neoadjuvant	Atezolizumab	642
			Control	644
Nishio et al., 2021 [38]	Lung cancer	First-line	Atezolizumab	291
			Control	274
Powles et al., 2020 [39]	Urothelial	First-line	Avelumab	349
			Control	342
Powles et al., 2021 [40]	Urothelial	First-line	Pembrolizumab	344
			Control	345
Pujade-Lauraine et al., 2021 [41]	Ovarian cancer	Second or later	Avelumab	182
			Control	177
Reck et al., 2016 [42]	Lung cancer	First-line	Ipilimumab	562
			Control	561
Rha et al., 2023 [43]	Gastro	First-line	Pembrolizumab	785
			Control	787
Rudin et al., 2020 [44]	Lung cancer	First-line	Pembrolizumab	223
			Control	223
Schmid et al., 2018 [45]	Breast cancer	First-line	Atezolizumab	460
			Control	430
Schmid et al., 2020 [46]	Breast cancer	First-line	Pembrolizumab	781
			Control	389
Shitara et al., 2020 [47]	Gastric cancer	First-line	Pembrolizumab	250
			Control	244
Socinski et al., 2018 [48]	Lung cancer	First-line	Atezolizumab	393
			Control	394
Song et al., 2023 [49]	Esophageal	First-line	Serplulimab	368
			Control	183
Sugawara et al., 2021 [50]	Lung cancer	First-line	Nivolumab	273
			Control	275
Usmani et al., 2019 [51]	Multiple myeloma	First-line	Pembrolizumab	154
			Control	148
Wang et al., 2022 [52]	Esophageal	First-line	Toripalimab	257
			Control	257
West et al., 2019 [53]	Lung cancer	First-line	Atezolizumab	473
			Control	131
Xu et al., 2022 [54]	Gastro	First-line	Sintilimab	328
			Control	320

**Table 2:** Quality assessment of the included studies

Study	Randomization process	Deviations from the intended interventions	Missing outcome data	Measurement of outcome	Selection of the reported result	Overall risk of bias
Cortes et al., 2020 [18]	Low	Low	Low	Low	Low	Low
Doki et al., 2022 [19]	Low	Low	Low	Low	Low	Low
Galsky et al., 2020 [20]	Low	Low	Low	Low	Low	Low
Gandhi et al., 2018 [21]	Low	Low	Low	Low	Low	Low
Govindan et al., 2017 [22]	Low	Low	Low	Low	Low	Low
Gutzmer et al., 2020 [23]	Low	Low	Low	Low	Low	Low
Hodi et al., 2010 [24]	Low	Low	Low	Low	Low	Low
Horn et al., 2018 [25]	Low	Low	Low	Low	Low	Low
Janjigian et al., 2021 [26]	Some concerns	Low	Low	Low	Low	Some concerns
Jotte et al., 2020 [27]	Low	Low	Low	Low	Low	Low
Kang et al., 2022 [28]	Low	Low	Low	Low	Low	Low
Kelly et al., 2023 [29]	Low	Low	Low	Low	Low	Low
Kwon et al., 2014 [30]	Low	Low	Low	Low	Low	Low
Lee et al., 2021 [31]	Low	Low	Low	Low	Low	Low
Lu et al., 2022 [32]	Low	Low	Low	Low	Low	Low
Luo et al., 2021 [33]	Low	Low	Low	Low	Low	Low
Mateos et al., 2019 [34]	Some concerns	Low	Low	Low	Low	Some concerns
Miles et al., 2021 [35]	Low	Low	Low	Low	Low	Low
Mittendorf et al., 2020 [36]	Low	Low	Low	Low	Low	Low
Moore et al., 2021 [37]	Low	Low	Low	Low	Low	Low
Nishio et al., 2021 [38]	Low	Low	Low	Low	Low	Low
Powles et al., 2020 [39]	Low	Low	Low	Low	Low	Low
Powles et al., 2021 [40]	Some concerns	Low	Low	Low	Low	Some concerns
Pujade-Lauraine et al., 2021 [41]	Some concerns	Low	Low	Low	Low	Some concerns
Reck et al., 2016 [42]	Low	Low	Low	Low	Low	Low
Rha et al., 2023 [43]	Low	Low	Low	Low	Low	Low
Rudin et al., 2020 [44]	Low	Low	Low	Low	Low	Low
Schmid et al., 2018 [45]	Low	Low	Low	Low	Low	Low
Schmid et al., 2020 [46]	Low	Low	Low	Low	Low	Low
Shitara et al., 2020 [47]	Low	Low	Low	Low	Low	Low
Socinski et al., 2018 [48]	Low	Low	Low	Low	Low	Low
Song et al., 2023 [49]	Low	Low	Low	Low	Low	Low
Sugawara et al., 2021 [50]	Low	Low	Low	Low	Low	Low
Usmani et al., 2019 [51]	Some concerns	Low	Low	Low	Low	Some concerns
Wang et al., 2022 [52]	Low	Low	Low	Low	Low	Low
West et al., 2019 [53]	Low	Low	Low	Low	Low	Low
Xu et al., 2022 [54]	Low	Low	Low	Low	Low	Low

Anemia

In the NMA, none of the ICIs showed a statistically significant increase in the risk of anemia compared with control, as shown in Table 3 and Appendix B. Based on treatment ranking, ipilimumab demonstrated the most favorable safety profile for anemia, followed by toripalimab and pembrolizumab. In contrast, serplulimab and nivolumab ranked lowest with respect to anemia risk. The remaining agents, including atezolizumab, avelumab, camrelizumab, and sintilimab, showed intermediate rankings. Overall, the relative risk estimates were comparable across treatments, indicating broadly similar anemia risk compared with control. Significant heterogeneity was observed within the control versus Ipilimumab comparison, while other treatment comparisons showed no evidence of heterogeneity.

**Table 3:** Risk of anemia associated with ICI RR: risk ratio; CI: confidence interval; SUCRA: surface under the cumulative ranking curve

Drug name	RR (95% CI)	SUCRA score
Atezolizumab	1.07 (0.99 to 1.15)	0.3454
Avelumab	1.05 (0.95 to 1.17)	0.4259
Camrelizumab	1.06 (0.91 to 1.22)	0.4314
Ipilimumab	0.94 (0.84 to 1.06)	0.8736
Nivolumab	1.10 (0.96 to 1.26)	0.2568
Pembrolizumab	1.02 (0.95 to 1.09)	0.5779
Serplulimab	1.14 (0.97 to 1.34)	0.1619
Sintilimab	1.05 (0.93 to 1.18)	0.4481
Toripalimab	0.97 (0.84 to 1.12)	0.7615

Neutropenia

For neutropenia, several vs were associated with a higher risk compared with control, while others showed no clear difference, as shown in Table 4 and Appendix C. Treatment ranking indicated that ipilimumab had the most favorable safety profile for neutropenia, followed by avelumab and pembrolizumab. In contrast, toripalimab and serplulimab ranked lowest with respect to neutropenia risk. The remaining agents, including camrelizumab, sintilimab, and atezolizumab, demonstrated intermediate rankings. Overall, the relative effects varied across treatments, suggesting heterogeneity in neutropenia risk among ICIs. No significant heterogeneity was observed within any treatment comparison.

**Table 4:** Risk of neutropenia associated with ICI RR: risk ratio; CI: confidence interval; SUCRA: surface under the cumulative ranking curve

Drug Name	RR (95% CI)	SUCRA score
Atezolizumab	1.11 (1.02 to 1.21)	0.3444
Avelumab	0.99 (0.84 to 1.16)	0.7477
Camrelizumab	1.08 (0.92 to 1.27)	0.4572
Ipilimumab	0.95 (0.82 to 1.09)	0.8805
Nivolumab	1.15 (1.01 to 1.31)	0.2444
Pembrolizumab	1.02 (0.95 to 1.10)	0.6483
Serplulimab	1.18 (0.96 to 1.44)	0.2348
Sintilimab	1.05 (0.93 to 1.18)	0.5667
Toripalimab	1.24 (1.04 to 1.49)	0.1083

Thrombocytopenia

With respect to thrombocytopenia, most ICIs showed no clear difference in risk compared with control, as shown in Table 5 and Appendix C. Treatment ranking based on SUCRA values indicated that ipilimumab had the most favorable safety profile, followed by sintilimab and nivolumab. In contrast, toripalimab ranked lowest for thrombocytopenia risk, while atezolizumab and serplulimab also showed relatively less favorable rankings. The remaining agents demonstrated intermediate rankings. Overall, the findings suggest variability in thrombocytopenia risk across treatments, although estimates were largely comparable to those of the control. Significant heterogeneity was observed within the control versus atezolizumab comparison, while other comparisons showed no evidence of heterogeneity.

**Table 5:** Risk of thrombocytopenia associated with ICI RR: risk ratio; CI: confidence interval; SUCRA: surface under the cumulative ranking curve

Drug Name	RR (95% CI)	SUCRA score
Atezolizumab	1.25 (1.08 to 1.44)	0.1973
Avelumab	1.01 (0.79 to 1.31)	0.609
Camrelizumab	1.05 (0.75 to 1.48)	0.5319
Ipilimumab	0.88 (0.69 to 1.12)	0.8699
Nivolumab	1.00 (0.84 to 1.20)	0.6434
Pembrolizumab	1.09 (0.94 to 1.27)	0.4323
Serplulimab	1.21 (0.90 to 1.62)	0.295
Sintilimab	0.96 (0.79 to 1.16)	0.7386
Toripalimab	1.72 (1.17 to 2.54)	0.0242

Inconsistency

Global inconsistency was assessed using the design-by-treatment interaction model for all three outcomes. No evidence of global inconsistency was detected for thrombocytopenia (Q = 25.53, df = 18, p = 0.11), neutropenia (Q = 30.73, df = 24, p = 0.16), or anemia (Q = 39.23, df = 28, p = 0.077). As the network adopted a star-shaped topology with no closed loops across all outcomes, formal evaluation of between-design inconsistency was not possible.

Publication Bias

Publication bias was assessed using Egger's test for each of the three hematological outcomes, acknowledging that this approach was originally developed for pairwise meta-analysis and its application in NMA remains methodologically debated. For anemia, no evidence of publication bias was detected (p = 0.92). For thrombocytopenia, results were similarly reassuring (p = 0.11). For neutropenia, Egger's test indicated potential small-study effects (p = 0.02), suggesting that smaller studies may have preferentially reported larger effect estimates, and findings for this outcome should therefore be interpreted with caution. Given the star-shaped network topology and the limitations of formal statistical testing for publication bias in NMA, all conclusions regarding publication bias are considered exploratory and indicative rather than definitive.

Discussion

This NMA represents the comprehensive comparative assessment of HAEs across different ICIs using indirect treatment comparisons. By synthesizing evidence from 37 RCTs encompassing diverse malignancies and treatment regimens, our analysis provides clinically relevant insights into the relative hematological safety profiles of available ICIs. The findings demonstrate notable heterogeneity in hematological toxicity risk across different checkpoint inhibitors, with important implications for treatment selection and patient counseling.

Based on SUCRA values, ipilimumab ranked highest for safety across all three hematological outcomes examined - anemia, neutropenia, and thrombocytopenia - though the corresponding effect estimates were not statistically significant, and these rankings should therefore be interpreted with caution rather than as evidence of superior safety. This finding is particularly noteworthy given that ipilimumab, as a CTLA-4 inhibitor, operates through a distinct mechanism of action compared to PD-1/PD-L1 inhibitors. CTLA-4 blockade primarily affects the priming phase of T-cell activation in lymphoid organs, whereas PD-1/PD-L1 inhibition predominantly acts in peripheral tissues and the tumor microenvironment [3]. This mechanistic difference may partly explain the divergent hematological toxicity patterns observed, though the precise immunological basis requires further investigation.

The superior ranking of ipilimumab regarding hematological safety appears paradoxical given its well-documented association with severe immune-related adverse events affecting other organ systems, particularly the gastrointestinal tract and endocrine system [4]. This discrepancy underscores the organ-specific nature of immune-related toxicities and highlights the importance of comprehensive safety profiling rather than extrapolating risks across different adverse event categories. Clinicians should recognize that a favorable hematological safety profile does not necessarily translate to overall superior tolerability, and treatment decisions must consider the complete spectrum of potential adverse events.

Among PD-1 inhibitors, pembrolizumab demonstrated relatively favorable rankings for anemia and neutropenia, while nivolumab showed less favorable profiles, particularly for anemia. These differences between agents targeting the same immune checkpoint are intriguing and may reflect subtle variations in binding characteristics, pharmacokinetic properties, or patient populations studied in the included trials. The newer PD-1 inhibitors-toripalimab, serplulimab, camrelizumab, and sintilimab-showed variable safety profiles, with toripalimab ranking favorably for anemia but poorly for neutropenia and thrombocytopenia. These agents, predominantly developed and initially studied in Asian populations, may exhibit different safety characteristics related to genetic polymorphisms, baseline hematological parameters, or concomitant medications used in different geographic regions [11].

The pathophysiology of ICI-associated HAEs remains incompletely understood, and the following mechanistic considerations are hypothesis-generating rather than established. For anemia, proposed immune-mediated mechanisms include autoimmune hemolytic anemia, pure red cell aplasia, and bone marrow infiltration by activated immune cells, although the relative contribution of each pathway remains unclear [54-55]. The observed variability in hematological toxicity incidence across different ICI agents may reflect differences in the degree and specificity of checkpoint receptor engagement, though this hypothesis requires prospective mechanistic validation. These pathophysiological explanations are derived from case reports and small series rather than the RCT-level evidence synthesised in this review and should be interpreted accordingly.

Thrombocytopenia associated with ICIs, particularly immune-related thrombocytopenia (irTCP), represents a clinically significant concern given its potential for serious bleeding complications. Our finding that atezolizumab showed relatively higher thrombocytopenia risk aligns with previous meta-analytic evidence suggesting elevated risk with PD-L1 inhibitors, particularly when combined with cytotoxic chemotherapy [8]. The mechanism likely involves production of platelet-reactive autoantibodies that accelerate platelet destruction via splenic macrophages and inhibit megakaryocyte maturation in bone marrow [56]. Interestingly, recent research has demonstrated that platelets can directly uptake PD-L1 from tumor cells and present it on their surface, particularly in non-small cell lung cancer, potentially making them targets for immune-mediated destruction during PD-L1 blockade [57]. This mechanism may explain the heightened thrombocytopenia risk observed with certain PD-L1 inhibitors in our analysis.

Our findings complement and extend previous meta-analytic work examining ICI-related hematological toxicities. Ohashi and colleagues conducted a systematic review and meta-analysis of 29 RCTs, reporting estimated incidence rates of 36.5% for all-grade anemia, 29.7% for all-grade neutropenia, and 18.0% for all-grade thrombocytopenia in patients receiving ICIs combined with systemic therapy [58]. However, their analysis primarily compared ICI combination regimens versus control arms without cytotoxic agents, limiting insights into comparative safety between different checkpoint inhibitors. By employing NMA methodology, our study addresses this critical gap by providing head-to-head comparisons between multiple ICIs that have never been directly compared in randomized trials.

Recent observational studies have identified specific risk factors for ICI-associated hematological complications, including pre-existing cytopenias, concurrent myelosuppressive therapy, smoking status, elevated body mass index, and certain cancer types, particularly melanoma and lung malignancies [59-60]. Our analysis, while unable to perform individual patient-level subgroup analyses due to reliance on aggregate trial data, nonetheless provides valuable comparative information that can inform risk stratification strategies when combined with patient-specific risk factors.

The variability in hematological safety profiles across different ICIs demonstrated in this analysis has important practical implications for clinical decision-making. For patients with baseline cytopenias, significant bone marrow reserve compromise, or those receiving concurrent myelosuppressive therapies, selection of agents with more favorable hematological safety rankings-such as ipilimumab, pembrolizumab, or avelumab-may be prudent when clinically appropriate and efficacy profiles are comparable. Conversely, for patients requiring specific agents based on tumor type and efficacy data, our findings emphasize the importance of enhanced hematological monitoring protocols.

However, several limitations warrant acknowledgment. First, as a study-level meta-analysis utilizing aggregate data rather than individual patient information, we could not perform detailed subgroup analyses based on patient-specific characteristics such as baseline hematological parameters, performance status, prior treatment exposure, or genetic factors that may modify hematological toxicity risk. Second, the star-shaped network geometry with limited closed loops precluded robust assessment of local inconsistency through node-splitting analysis, potentially masking inconsistencies between direct and indirect evidence where they exist. Third, the detection of publication bias for neutropenia suggests possible overestimation of treatment effects for this outcome, necessitating cautious interpretation despite the overall robustness of findings.

Fourth, substantial heterogeneity in study designs, including variations in concomitant therapies, dosing schedules, tumor types, and treatment lines, may have influenced the observed differences between agents. While our random-effects models account for between-study heterogeneity statistically, clinical heterogeneity remains a potential confounding factor in interpreting comparative effects. Fifth, the relatively limited number of studies for some newer checkpoint inhibitors (toripalimab, serplulimab, camrelizumab, and sintilimab) may result in less precise effect estimates for these agents compared to more extensively studied drugs like pembrolizumab and nivolumab.

Sixth, our analysis focused exclusively on three common HAEs (anemia, neutropenia, thrombocytopenia) but did not capture rarer but potentially more severe complications such as aplastic anemia, hemophagocytic lymphohistiocytosis, or acquired hemophilia, which have been reported with ICIs but occur too infrequently for meta-analytic assessment [61]. Finally, the median follow-up duration varied across studies, and some late-onset hematological toxicities may not have been captured within the observation periods of included trials.

## Conclusions

This comprehensive network meta-analysis identified heterogeneity in HAE profiles across different ICIs. Ipilimumab appeared to rank favorably for anemia, neutropenia, and thrombocytopenia, while newer agents demonstrated variable profiles across different cytopenias. Among PD-1/PD-L1 inhibitors, pembrolizumab and avelumab showed relatively favorable hematological safety rankings. These findings suggest potential differences in hematological safety profiles between agents; however, given the network structure, reliance on indirect comparisons, and observed heterogeneity across studies, the results should be interpreted cautiously. While not definitive, the analysis may provide preliminary evidence to inform clinical decision-making, particularly when considering immunotherapy for patients with pre-existing hematological vulnerabilities or those receiving concurrent myelosuppressive treatments. The observed variability in toxicity risk may support consideration of agent-specific and risk-stratified monitoring approaches. As ICIs continue to expand into earlier disease stages and broader patient populations, further well-designed head-to-head studies are needed to better clarify their comparative hematological safety profiles.

## Figures and Tables

**Figure 5:**
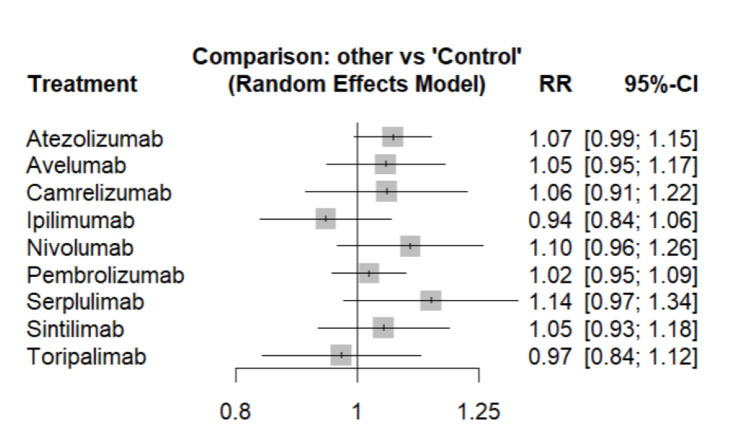
Forest plot of anemia

**Figure 6:**
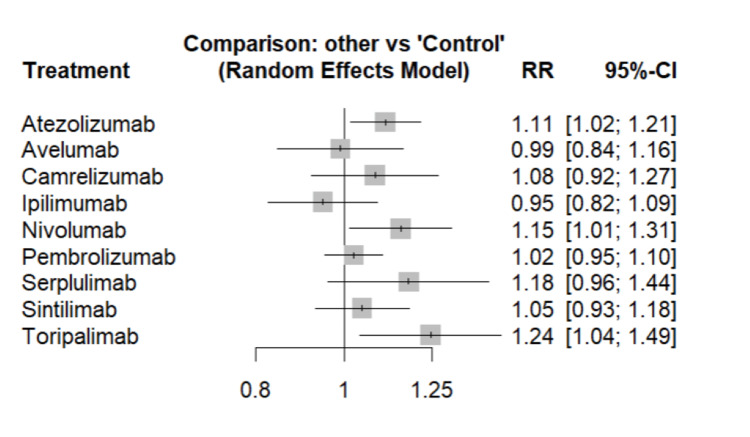
Forest plot of neutropenia

**Figure 7:**
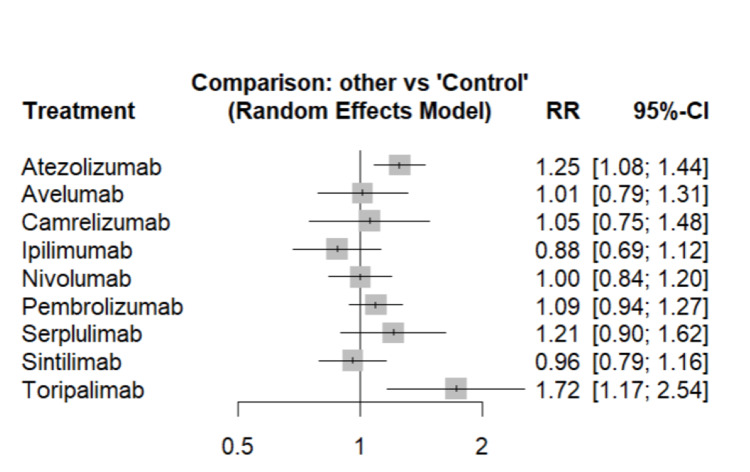
Forest plot of thrombocytopenia

## Appendices

Appendix A (for PubMed)

#1  (pembrolizumab[tiab] OR nivolumab[tiab] OR atezolizumab[tiab] OR durvalumab[tiab]

     OR avelumab[tiab] OR ipilimumab[tiab] OR cemiplimab[tiab] OR dostarlimab[tiab]

     OR "PD-1 inhibit*"[tiab] OR "PD-L1 block*"[tiab] OR "CTLA-4 inhibit*"[tiab]

     OR "immune checkpoint inhibitor*"[tiab] OR "immune checkpoint therap*"[tiab]

     OR "anti-PD-1"[tiab] OR "anti-PD-L1"[tiab] OR "anti-CTLA-4"[tiab]

     OR "checkpoint blockade"[tiab]

     OR "Programmed Cell Death 1 Receptor"[MeSH] OR "B7-H1 Antigen"[MeSH]

     OR "CTLA-4 Antigen"[MeSH] OR "Immune Checkpoint Inhibitors"[MeSH])

#2  ("hematologic adverse event*"[tiab] OR "haematologic adverse event*"[tiab]

     OR cytopenia*[tiab] OR anemia[tiab] OR anaemia[tiab] OR neutropenia[tiab]

     OR thrombocytopenia[tiab] OR leukopenia[tiab] OR leucopenia[tiab]

     OR pancytopenia[tiab] OR "hematological toxicit*"[tiab]

     OR "haematological toxicit*"[tiab] OR "bone marrow suppression"[tiab]

     OR Anemia[MeSH] OR Neutropenia[MeSH] OR Thrombocytopenia[MeSH]

     OR Leukopenia[MeSH] OR Pancytopenia[MeSH]

     OR "Hematologic Diseases"[MeSH])

#3  ("randomized controlled trial"[pt] OR "controlled clinical trial"[pt]

     OR randomized[tiab] OR randomised[tiab] OR "random allocation"[tiab]

     OR "randomly assigned"[tiab] OR placebo[tiab] OR "double-blind"[tiab]

     OR "double blind"[tiab] OR "clinical trial"[pt]

     OR "Randomized Controlled Trials as Topic"[MeSH])

#4  #1 AND #2 AND #3

Appendix B

Appendix C 

Appendix D 
